# Extracellular matrix-inspired biomaterials for wound healing

**DOI:** 10.1007/s11033-024-09750-9

**Published:** 2024-07-22

**Authors:** Louise Hosty, Thomas Heatherington, Fabio Quondamatteo, Shane Browne

**Affiliations:** 1 Tissue Engineering Research Group, Department of Anatomy and Regenerative Medicine, Royal College of Surgeons in Ireland, 123, St Stephen’s Green, Dublin 2, Ireland; 2 CÙRAM, Centre for Research in Medical Devices, University of Galway, Galway, H91 W2TY Ireland; 3 Trinity Centre for Biomedical Engineering, Trinity College Dublin, Dublin 2, Ireland

**Keywords:** Diabetic foot ulcer, Extracellular matrix, Biomaterials, Wound healing, Decellularisation

## Abstract

Diabetic foot ulcers (DFU) are a debilitating and life-threatening complication of Diabetes Mellitus. Ulceration develops from a combination of associated diabetic complications, including neuropathy, circulatory dysfunction, and repetitive trauma, and they affect approximately 19–34% of patients as a result. The severity and chronic nature of diabetic foot ulcers stems from the disruption to normal wound healing, as a result of the molecular mechanisms which underly diabetic pathophysiology. The current standard-of-care is clinically insufficient to promote healing for many DFU patients, resulting in a high frequency of recurrence and limb amputations. Biomaterial dressings, and in particular those derived from the extracellular matrix (ECM), have emerged as a promising approach for the treatment of DFU. By providing a template for cell infiltration and skin regeneration, ECM-derived biomaterials offer great hope as a treatment for DFU. A range of approaches exist for the development of ECM-derived biomaterials, including the use of purified ECM components, decellularisation and processing of donor/ animal tissues, or the use of in vitro-deposited ECM. This review discusses the development and assessment of ECM-derived biomaterials for the treatment of chronic wounds, as well as the mechanisms of action through which ECM-derived biomaterials stimulate wound healing.

## Introduction

Diabetes mellitus (DM) is a systemic metabolic disorder which is characterised by hyperglycaemia [1]. Over half a billion people are living with DM worldwide, and the number of patients is projected to increase substantially to over a billion by 2050, thus worsening the social and economic burden of DM and its associated complications [2]. Type 1 diabetes mellitus (T1DM) is an autoimmune condition which is characterised by the destruction of pancreatic insulin-producing β-cells, leading to hyperglycaemia [3]. In contrast, the onset of insulin resistance in T2DM typically occurs as a result of poor lifestyle and hyperglycaemia, leading to the onset of diabetic complications [3, 4]. Diabetic complications can be debilitating and life-threatening, and include retinopathy, nephropathy, neuropathy, cardiovascular dysfunction and chronic wounds [5].

As a result of DM-associated vascular complications, wound healing capacity in DM patients is also severely affected and can lead to the formation of chronic wounds. Diabetic foot ulcers (DFU) are particularly prevalent, affecting approximately 19–34% of DM patients [6]. Ulceration develops from a combination of neuropathy, circulatory dysfunction, and trauma [7]. Anatomical deformities including the claw toe, are particularly susceptible to the development of DFUs and chronic wounds, as a result of DM-induced neuropathy, and areas of high pressure and repetitive trauma in long-term diabetic patients [8, 9]. Due to the non-healing nature of DFUs, and the consistent exposure of the open wound to the environment, microbial infection and severe infection with bone involvement or osteomyelitis, are major risks to patients. Osteomyelitis can ultimately lead to the need for limb amputations, frequently seen in the lower limb [10]. The current standard of care for DFU includes rigorous protocols of debridement, off-loading, and antibiotics to treat infection [11]. However, standard wound-care has proven insufficient for many patients, as 20% of DFU patients eventually require lower limb amputations [6]. Despite the lower severity of DFUs in comparison to other fatal diabetic complications, it remains an urgent global concern due to its associated economic and social burden, significant impact on quality of life, and ever-growing frequency. As such, it is evident that there is an urgent need for the improvement of treatment strategies for DFUs [12].

In recent years, the use of biomaterials in wound healing applications has shown great promise to treat chronic wounds. Biomaterials are classed as either composed of synthetic or naturally-derived materials, either of which may be processed or modified to further enhance their wound healing capacity [13]. These alterations can include chemical modification to control biophysical properties and/or bioactivity [14]. While a wide range of biomaterials have been developed to promote tissue regeneration, biomaterials derived from the extracellular matrix (ECM) have shown particular promise [15]. Owing to their inherent biocompatibility, biodegradation, and minimal immunogenicity, host cells will readily populate and remodel ECM-derived materials [16]. This review focuses on the development of ECM-derived biomaterial systems, and their capacity to enhance repair and regeneration in the context of DFUs. Furthermore, we will discuss the mechanisms of action of ECM-derived biomaterials, and finally point towards promising developments in the field that can lead to the clinical realisation of ECM-derived biomaterials as efficacious therapies for patients.

### The molecular basis of diabetic foot ulcers

The onset of DFU is a multifactorial process which is stimulated by repetitive un-noticed trauma, and exacerbated by poor wound healing, peripheral neuropathy, and cardiovascular dysfunction. In DM-induced neuropathy, hyperglycaemia leads to an overproduction of reactive oxygen species (ROS), mitochondrial failure, and subsequent oxidative damage to neurons and Schwann cells [17]. Both motor and sensory neurons are affected in the process, thus leading to muscle atrophy, anatomical deformities, and a loss of sensation. Decreased sensation in the lower limbs can lead to patients ignoring or not noticing repeated trauma and the formation of a wound. This can lead to the development of a chronic wound or DFU over time [7].

**Fig. 1:**
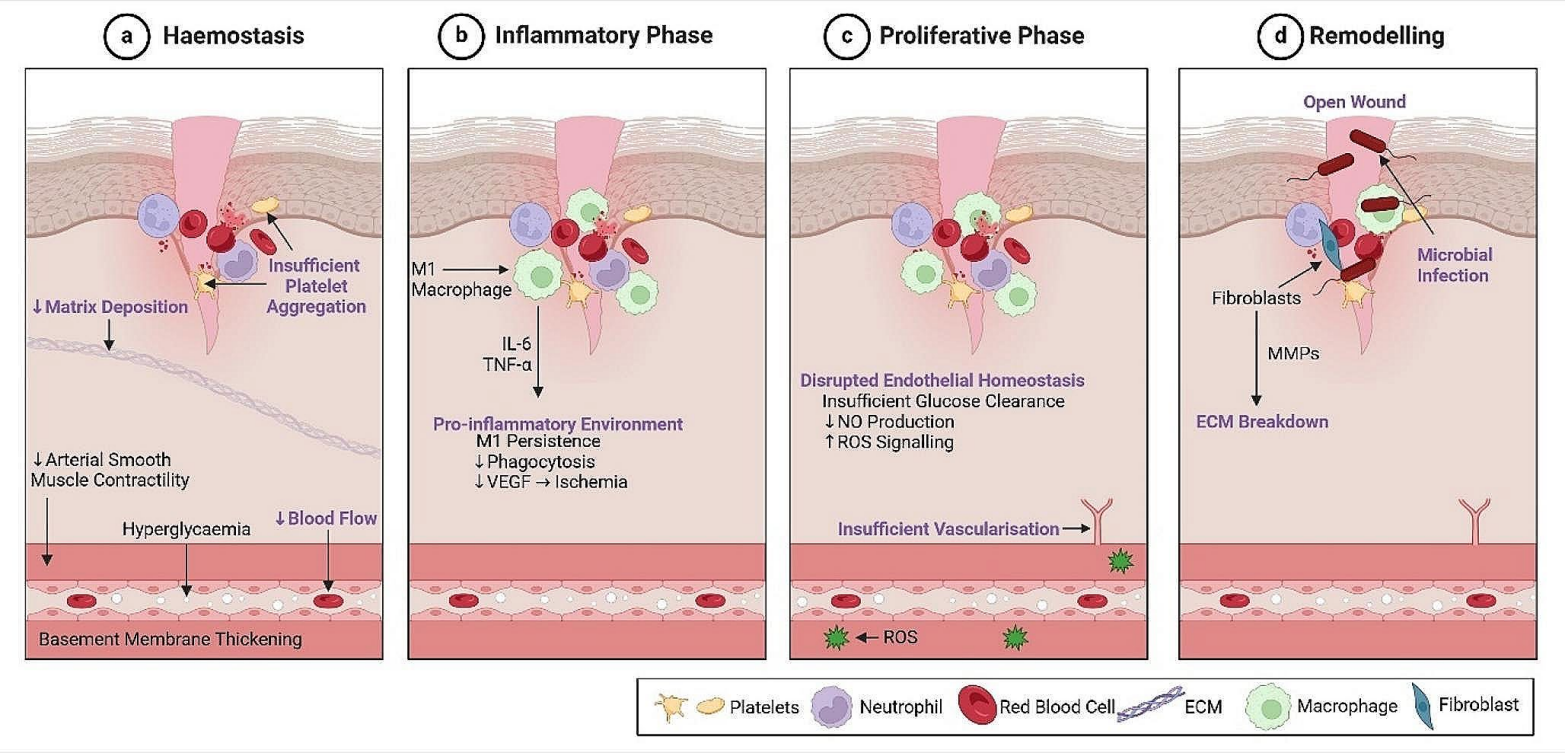
Disruption to the phases of wound healing in diabetic patients. The 4 phases of wound healing are negatively affected in diabetic patients. **a**. During Haemostasis, thickening of the blood vessel walls and reduced contractility of smooth muscle leads to perturbed vasoconstriction and reduced blood flow. Subsequent ischemia leads to insufficient platelet aggregation which prevents progression of blood coagulation after injury and delays healing. Reduced deposition of ECM at this stage also delays healing. **b**. The inflammatory phase is prolonged, as seen by pro-inflammatory macrophage (M1) persistence. Secretion of pro-inflammatory cytokines e.g., IL-6 and TNF-a leads to reduced phagocytosis and VEGF secretion, thus delaying angiogenesis and exacerbating local ischemia. **c**. As a result of insulin resistance and disrupted endothelial homeostasis in the previous phases, there is reduced nitric oxide and increased ROS production within the endothelial membrane, further preventing angiogenesis/ vasculogenesis. **d**. Finally, tissue remodelling is inhibited through insufficient progression of healing. Fibroblasts within a wound release excessive MMPs which are disruptive to ECM components including collagens. Similarly, exposure of the open wound to the environment increases susceptibility to serious infection during healing. Figure was created in Biorender

Classical wound healing involves 4 key stages: haemostasis, inflammation, proliferation, and remodelling. These processes involve a highly regulated and balanced interplay between inflammation, cell migration and angiogenesis, and tissue regeneration, leading to ECM deposition, remodelling, and healing. For an in-depth description and discussion of these processes under physiological conditions, the reader is referred to a number of excellent papers [18–21]. While the classical wound healing process ultimately leads to wound closure and functional restoration of the skin in those without underlying conditions, this is not the case for patients with DM. In diabetic patients, many of the key stages of wound healing are dysfunctional due to hyperglycaemia, neuropathy, ischemia and chronic inflammation [22]. Figure 1 depicts a schematic of the phases of wound healing, and how they become disrupted in diabetic patients.

Further, hyperglycaemia-induced kidney damage contributes to the onset of raised blood pressure and atherosclerosis. This reduces vessel contractility, impeding vasodilation and reducing blood flow. Ultimately, reduced blood flow and thickened capillary membranes inhibit perfusion of peripheral tissue and impede wound repair i.e., a DFU [4].

The first stage of wound healing impacted by DM is haemostasis. Capillary basement membrane thickening, and reduced contractility of arterial smooth muscle, in diabetic patients, diminishes vasoconstriction, obstructing healing from the outset [23]. Platelet plug formation is disrupted as a result of this ischemia, further delaying the coagulation cascade and lowering clot stability. Ultimately, there is less provisional matrix deposition during this phase, and subsequently reduced cellular invasion [24].

In DFUs, the inflammatory phase is prolonged, leading to a pro-inflammatory micro-environment that inhibits regeneration. This includes the secretion of pro-inflammatory cytokines and proteins, including interleukin (IL)-1 and − 6, and tumour necrosis factor alpha (TNF-α) [25, 26]. The persistence of pro-inflammatory (M1) macrophages is coupled with their reduced phagocytosis of microbes and efferocytosis of apoptotic cells and debris, further preventing cessation of the pro-inflammatory phase [27, 28]. In classical wound healing, a transition from M1 to anti-inflammatory (M2) macrophages occurs, promoting resolution of inflammation and tissue vascularisation. However, in DFUs, this transition does not occur, thus hindering vascularisation and angiogenesis, amplifying ischemia, and preventing wound healing [18].

The proliferative phase of wound healing is typically characterised by increased tissue vascularisation, the formation of granulation tissue, and ECM deposition by fibroblasts [22]. Similarly, angiogenesis and the formation of new blood vessels is a critical step of wound healing, which normally facilitates the restoration of endothelial and tissue homeostasis, through antioxidant signalling [18, 29]. However, hyperglycaemia leads to endothelial dysfunction and the inhibition of tissue vascularisation. This includes reduced nitric oxide (vasodilator and vasoprotector) production, and increased reactive oxygen species (ROS) generation [29, 30]. High levels of ROS signalling further contributes to the perturbation of vascularisation and a reduction in the formation of blood vessels [18].

Persistence of a pro-inflammatory and ischemic microenvironment in DM further affects the remodelling phase of wound healing through elevated production of matrix metalloproteinases (MMPs), driving the degradation of ECM components [31]. ECM deposition and collagen fibre reorganisation are essential for tissue repair and the re-establishment of the structural integrity and barrier function of the skin, which includes proper epithelial cell differentiation [32, 33]. As such, excessive MMP activity and ECM degradation inhibits re-epithelisation, and results in the wound remaining open [33]. Furthermore, prolonged exposure of the open wound to the environment increases the risk of infection and biofilm formation, further complicating and hindering repair [34].

Clinically it has been observed that even a DFU that achieves re-epithelisation, is highly likely to recur due to the suboptimal mechanical characteristics of the neotissue [35]. The current rate of recurrence is approximately 42% over 1 year, increasing to 65% after 5 years [36]. Consequently, this leads to the high frequency of amputations as a preventative measure against wound recurrence and systemic infection [37]. However, even after limb amputations, there is a high frequency (~ 43%) of surgical wound complications among patients due to the underlying, untreated pathophysiology [38]. The following section details the current standard of care for the treatment of DFU.

## Established therapies for DFUs

The current standard of care for DFUs aims to support wound healing through regular debridement, wound dressings, and pressure off-loading [22]. The removal of necrotic tissue by debridement aids healing and skin regeneration through the elimination of infection, and the removal of the physical barrier for cell migration [11]. Wound dressings and topical solutions including antibiotics or antimicrobial ointments are applied, and regularly changed to maintain a moist environment and prevent infection [22, 39], while pressure off-loading, and the use of a total-contact casts or a boot limits ambulation and prevents DFU aggravation [40]. Other approaches include negative pressure wound therapy (NPWT) and hyperbaric oxygen therapy, which are recommended as adjunctive therapies to other treatments for the removal of infection and promotion of vascularisation [41, 42]. Skin grafts have also shown promise, but their invasive nature coupled with a considerable risk of infection, makes them a less ideal solution [43]. Similarly, the underlying DM pathophysiology increases the likelihood of graft failure [39].

Current therapies predominantly aim to protect wounds from infection and rely on the inherent regeneration capacity of the tissue for wound closure to occur. However, owing to the limited regenerative potential and healing capacity of DFUs, these treatments are insufficient for many diabetic patients [44]. Given that the number of diabetic patients worldwide is rapidly increasing, there is a definitive need for more advanced therapeutic options [45]. The next section will describe how biomaterials have emerged as a promising therapeutic approach, acting as a template to support cell infiltration and neotissue formation, accelerating repair.

## Biomaterials as an emerging DFU therapy

**Fig. 2:**
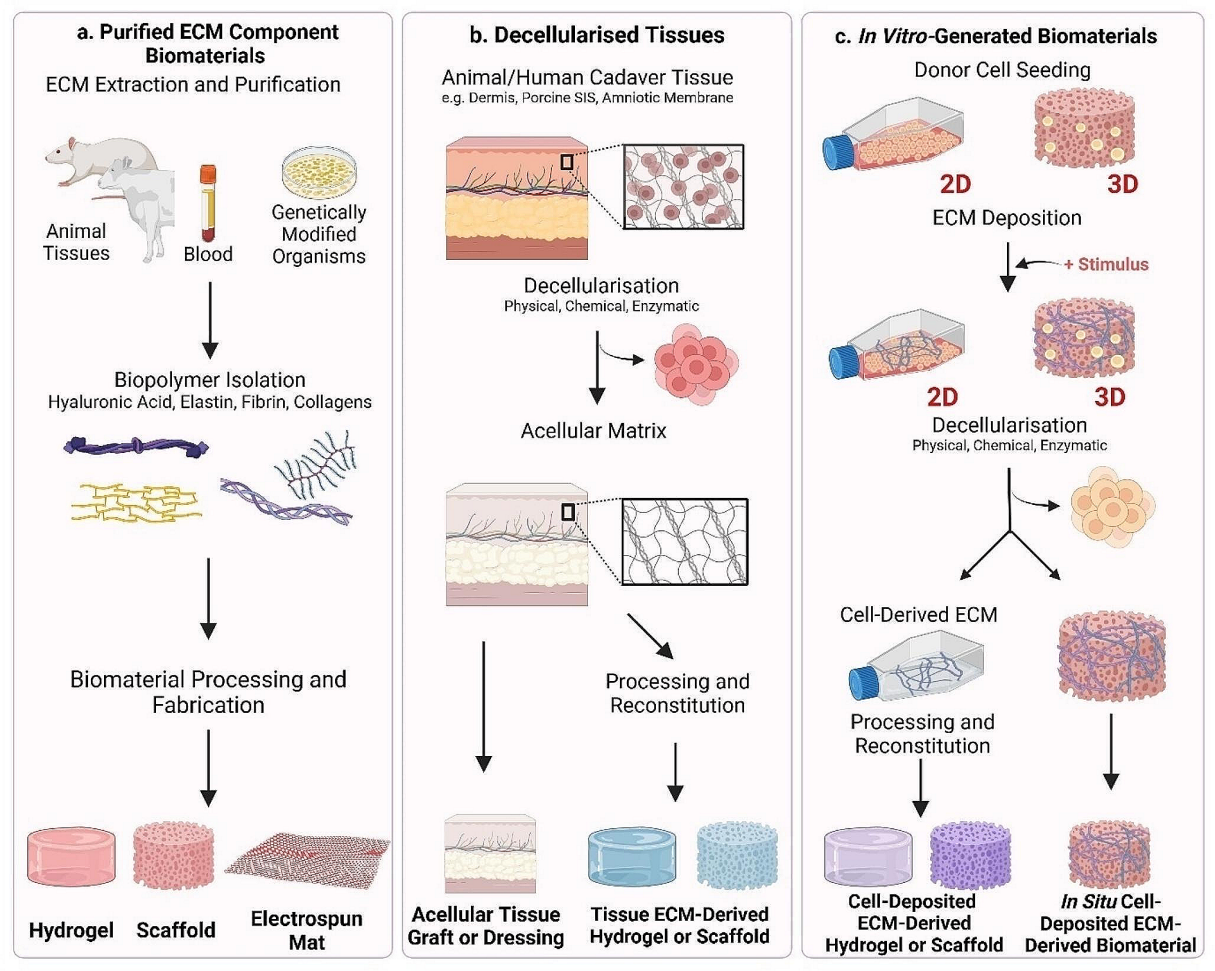
ECM-Derived Biomaterials Production and Application. There are 3 principal production methods for ECM-derived biomaterials, as discussed throughout this review. Purified ECM components, decellularised tissues, and in vitro-generated ECM are then processed to produce a biomaterial for DFU treatment. **a**. Purified ECM Component Biomaterials are produced from isolated ECM components including collagen, fibrin, hyaluronic acid, and elastin. ECM-derived biomaterials can be produced from single components, or from a combination of components e.g. collagen and hyaluronic acid. **b**. Decellularised tissue-derived ECM biomaterials are produced from animal or cadaver tissue, and decellularised to produce an acellular matrix. **c**. In vitro-generated biomaterials can be produced in 2 ways. Firstly, cells are kept in culture as they deposit their ECM over time. Decellularisation then removes the cellular components and preserves their ECM. Secondly, cells are seeded to a biomaterial and allowed to deposit their ECM over time, which is optimised through induction of a stimulus e.g., macromolecular crowding, ascorbic acid, increased cell density. Decellularisation then removes the cellular component, and preserves the deposited ECM, leaving an in situ deposited ECM-derived scaffold which can be implanted to an ulcer. Figure was created in Biorender

By providing a provisional matrix for cell migration, infiltration, and proliferation, biomaterials can support neotissue formation and healing [13, 46–48]. A number of biomaterials have been assessed in clinical trials as an alternative to standard wound care protocols and have demonstrated great potential. For example, the ‘Integra Dermal Regeneration Template’ (IDRT), a bi-layered collagen/ chondroitin-6-sulphate matrix, has shown significant promise to promote DFU healing [48]. Similarly, Alloderm™, a cadaveric skin-derived dermal substitute is FDA-approved for treatment of DFUs [49]. These ECM-derived biomaterials, in addition to many others at the stage of pre-clinical and in vitro assessment, emphasise the great promise that biomaterials hold to promote wound repair. A schematic summary of the production and application of ECM-derived biomaterials is provided in Fig. 2.

Biomaterials are typically classed as either of natural or synthetic origin. Although synthetic biomaterials have proven useful in tissue engineering due to their ease of manufacture and modification, natural biomaterials are more widely used owing to a number of reasons [50]. In particular, mammalian ECM-derived biomaterials are of significant interest due to their inherent biocompatibility, bioactivity, and biodegradation, as well as the presence of cell-recognition motifs [16, 51]. In fact, ECM-derived biomaterials have shown particular promise for tissue regeneration in a wide range of applications, including wound repair [13]. ECM-derived biomaterials may be of allogeneic or xenogeneic origin, and their production includes the extraction and processing of distinct ECM components, decellularisation of whole human or animal tissue, or the processing of ECM deposited in vitro [52]. The following sections will describe these techniques in more detail.

### Purified components

Biomaterials composed of isolated ECM components constitute the simplest form of ECM-derived biomaterials for tissue repair. Typically, ECM-derived biopolymers such as collagen, hyaluronic acid (HyA), fibrin, and gelatin, are chemically or physically crosslinked to form a structure to facilitate cell infiltration and tissue repair. These biopolymers are particularly suitable for wound repair owing to their presence in the native ECM, as well as their inherent biocompatibility, biodegradability, and low immunogenicity [52, 53].

Collagen type I is the most abundant component of the ECM and has been widely used as a biomaterial due to its established capacity to facilitate cell migration, infiltration and proliferation [54, 55]. Collagen-based biomaterials have been investigated in a wide variety of forms for the treatment of DFUs, including scaffolds, gels, particles, and films. For example, collagen type I electrospun matrices demonstrated enhanced keratinocyte adhesion, implying its potential to support wound healing [56]. Furthermore, fish skin-derived collagen was electrospun to form a nanofibrous mesh, and facilitated adhesion, proliferation, and differentiation of human keratinocytes in vitro. This further demonstrated the biocompatibility and pro-regenerative capacity of electrospun collagen in the context of wound re-epithelialisation, which was shown in a murine model [57]. Collagen-based biomaterials can also support cell delivery, as demonstrated when mesenchymal stem cells (MSCs) seeded in collagen type I scaffolds enhanced healing in rabbit DFU models, through increased angiogenesis and accelerated wound closure [58]. Further pre-clinical studies which have shown the efficacy of collagen-based biomaterials in wound healing include the Promogran^®^ sponge, made from collagen and oxidised regenerated cellulose. This combination of oxidised regenerated cellulose and collagen has previously shown to be effective for the improvement of haemostasis and the treatment of intraoperative bleeding, further aligning with its efficacy in wound healing [59]. In Promogran^®^-treated DFUs, the sponge displayed inhibitory effects on protease-driven pathways, thus impeding protease-driven growth factor/ECM degradation, accelerating re-epithelialisation, and shortening healing times, in comparison to commercial hydrocolloid-treated wounds [60]. Similarly, the use of lyophilised collagen particles as a wound dressing has effectively reduced DFU wound size by 45.43%, in comparison to 23.40% of control hydrocolloid dressing-treated wounds [61].

Hyaluronic acid (HyA) is a naturally occurring ECM-derived biopolymer, whose inherent ability to promote wound healing, and regulate inflammation, angiogenesis, and cell migration is well-documented [62–65]. Furthermore, HyA has been widely used to develop biomaterials, in particular hydrogels with great potential for tissue repair [66–70]. The hydrophilic nature of HyA is also of crucial importance in the development of a wound healing therapeutic, due to its ability to maintain a moist wound environment, facilitating epithelial migration [71, 72]. Like collagens, a variety of forms of HyA-derived biomaterials have been assessed for their wound healing capacity, including electrospun scaffolds and hydrogels. Dermal fibroblast infiltration has been seen throughout HyA-based scaffolds in a porosity-dependent manner, implying its suitability as a template for tissue integration and wound healing. The implantation of these electrospun HyA scaffolds in full-thickness mouse wounds also facilitated increased vascularisation and re-epithelialisation in comparison to silicon dressings [73]. Hyaff-11 (a HyA-based wound dressing) has also promoted effective ECM deposition and endothelial cell proliferation in vitro, with up to 50% reduction in DFU wound size, increased endothelial cell proliferation, and ECM deposition (collagens, fibronectin and laminins) in vivo [64, 74, 75].

Fibrin is the primary protein component of blood clots and has been assessed as a biomaterial to drive DFU repair [19]. Its success in wound healing therapies can be partially attributed to the role that it plays in the early stages of wound healing, particularly the formation and stabilisation of the fibrin clot, which acts as a template for cell infiltration [76]. Owing to its pro-regenerative capacity, it has been extensively used in wound healing, with fibrin hydrogels demonstrating immunomodulatory effects on macrophages in vitro, thus driving the resolution of the inflammation [77]. Similarly, fibrin biomaterials have laid the foundation for the development of many hybrid scaffolds. For example, fibrin hydrogels and electrospun membranes (with poly(lactide-co-glycolide)) have been combined to develop a bi-layered scaffold. After implantation in a full-thickness rat wound model, these scaffolds stimulated re-epithelialisation and deposition of collagens, demonstrating wound healing potential [78]. Platelet rich plasma (PRP) which is the fluid component of blood, can be obtained through centrifugation, contains higher amounts of platelets than blood, and is frequently used therapeutically e.g., cartilage repair surgery [79, 80]. Additionally, platelet rich fibrin (PRF), is a PRP-derivative which is rich in platelets, leukocytes, and growth factors within a fibrin matrix. The autologous nature and abundance of growth factors in both blood derivatives make them good candidates for the production of a wound healing biomaterial [81]. In DFU clinical trials, hybrid PRP/PRF scaffolds facilitated significant reduction in wound size in both diabetic and non-diabetic treatment groups [82]. A similar study by Game et al. demonstrated full wound closure in 34.1% of PRF-patch treated DFU patients, in comparison to 22% in the standard care control groups [83].

The inclusion of fibronectin in biomaterials has also been assessed for wound healing applications. Fibronectin meshes were produced by rotary jet spinning and were applied to full-thickness mouse wounds with polymer films as adhesives. The fibronectin meshes increased wound closure and epidermal thickness in comparison to non-treated wounds over 16 days [84]. Gelatin, or hydrolysed collagen, is another product of the ECM which has also been used as a biomaterial for wound healing. Owing to its bioactivity, biodegradability, and easily modifiable nature, it is particularly suitable for wound healing [85]. Gelatin hydrogels have been used as cell-delivery vehicles for wound healing, with laminin and fibronectin deposition shown in vitro, and accelerated wound closure, re-epithelialisation, and vascularisation in preclinical mouse models [86].

Although collagen, HyA, fibrin, fibronectin, and gelatin-based biomaterials have demonstrated promise, more complex biomaterials involving combinations of multiple ECM components may be necessary to drive wound healing. When used in combination, these biomaterials constitute a more complex structure which more faithfully recapitulates the native ECM. This can be seen in collagen-based biomaterials, which have been widely assessed in combination with a range of other ECM components. For example, collagen type I and HyA scaffolds have facilitated increased fibroblast attachment and proliferation in vitro [87]. Similarly, collagen type I-HyA hydrogels have facilitated both endothelial cell and fibroblast attachment, infiltration, and proliferation in vitro, further supporting their potential in wound healing. This was also confirmed in preclinical models, with the highest reduction in wound size after collagen type I-HyA hydrogel treatment [88]. Similarly, the IDRT is a bi-layered matrix which incorporates both collagen type I and chondroitin-6-sulphate. Application of IDRT to DFUs, traumatic wounds, and life-threatening burns has supported a significant decrease in wound size, and accelerated wound closure [48, 89–93].

Elastin has also been used in combination with collagens for wound dressings which have increased flexibility, supporting coverage of curved surfaces such as the toes [57]. In a 2013 clinical study, elastin/ collagen type I scaffolds demonstrated significantly faster wound healing, resulting in shorter hospital stays [94]. Further optimisation of collagen type I/elastin scaffolds, including pore alignment and cross-linking, has also enhanced their wound healing potential through increased fibroblast proliferation [95]. The wound healing potential of a collagen type III/elastin mesh was further substantiated in a case report of full wound closure after traumatic tissue loss in an elderly diabetic patient [96]. Sulphated HyA, chondroitin sulphate, and gelatin have also been combined to form electrospun scaffolds, which mimicked the biological and mechanical cues of native skin. This led to increased proliferation of fibroblasts, keratinocytes, and human MSCs [97]. Finally, clinical evaluation of the pro-healing capacity of PRF/HyA hydrogels demonstrated increased VEGF and decreased IL-6 expression, implying enhanced healing through angiogenesis and reduced inflammation [98].

ECM-derived biomaterials constitute a simple but promising approach to support healing of diabetic wounds, a summary of which is presented in Table 1. However, despite demonstrating clear promise, it is evident that the complexity of the ECM is not fully recapitulated in biomaterials composed of only a small number of ECM components. It is likely that alternate approaches are necessary that can more faithfully recapitulate the composition, complexity and functionality of the native ECM.

**Table 1:** Summary table of purified ECM component biomaterials

Biomaterial Components	Form Factor	In Vitro Characterisation	In Vivo Model	In Vivo Outcome	Reference
Collagen, ECM protein coating (collagen type 1 or laminin or fibronectin)	Electrospun Scaffold	Increased keratinocyte adhesion and spreading with collagen type 1 and laminin coating	Full-thickness, non-diabetic rat wound model	Enhanced capillary density, fibroblast proliferation, and dense connective tissue after 1 week in collagen type 1-coated scaffold Epithelialisation complete after 1 week in both	[56]
Collagen	Electrospun Scaffold	No proliferation of mouse lymphocytes Increased keratinocyte adhesion, proliferation, and differentiation	Full-thickness, non-diabetic rat wound model	No significant activation of immune response Enhanced wound healing after 7 days Increased epithelialisation	[57]
Collagen	Scaffold	High cell infiltration to scaffold at high mesenchymal stromal cell (MSC) seeding density	Alloxan-induced hyperglycaemic rabbit wound model	Enhanced wound closure with higher MSC density Reduced radial diffusion No significant increase in inflammatory cells	[58]
HyA	Scaffold	Attachment and proliferation of endothelial cells Deposition of endothelial matrix in compacted scaffolds	N/A	N/A	[74]
Gelatin, PEG	Hydrogel	Enhanced adipose-derived stem cell (ASC) viability, proliferation, migration, and network formation when encapsulated Enhanced stemness and wound healing gene expression at high seeding density	Stented excisional, splinted wound, non-diabetic mouse model	Accelerated healing, hydrogel degradation, host cell infiltration remodelling, and vascularisation with ASCs No immune response to degradation products	[86]
HyA, fibrinogen, collagen, gelatin, chondroitin sulphate	Fibrous Hydrogel	Higher porosity facilitated infiltration of fibroblasts	Full-thickness, splinted non-diabetic mouse models	Accelerated wound closure and re-epithelialisation with higher porosity Increased vascularisation, Increased scaffold degradation and biodegradation	[73]
Gelatin, HyA/ sulphated HyA, Chondroitin-4-sulphate	Scaffold	Increased proliferation and adhesion of fibroblasts, keratinocytes, mesenchymal stem cells	N/A	N/A	[97]
Collagen, HyA	Scaffold	No fibroblast toxicity, supported attachment and proliferation	Non-diabetic rabbit intramuscular implantation	Capsule formation, neovascularization, tissue ingrowth and cell infiltration.	[87]
Fibronectin	Rotary Jet-Spun Scaffold	N/A	Full-thickness wounds in C57BL/6 mice	Accelerated wound closure Similar skin morphology Restored epidermal thickness Restored lipid layer Enhanced hair follicle and sebaceous gland recovery	[84]
Fibrin-Alginate	Scaffold	N/A	Full-thickness porcine wound model	Reduced wound area and contraction	[99]

### Decellularised tissue

Decellularised ECM (dECM) consists of whole tissues from animal or human cadavers, which are subsequently treated to remove the cellular components and retain the native ECM structure [100]. A number of physical, chemical and enzymatic protocols have been developed to remove cells and ensure that the remaining ECM retains its complex composition, architecture, and bioactivity [100]. This class of biomaterial aims to provide a template for native cell infiltration and stimulate wound repair, a summary of which can be seen in Table 2. Although the structure of the tissue can be retained in these decellularisation processes, the material may also be reconstituted into other forms including lyophilised scaffolds, injectable hydrogels or electrospun structures [101]. Commonly used tissues for the production of decellularised tissue-derived biomaterials include the dermis, placenta, and porcine small intestinal submucosa (SIS) [9].

**Table 2:** Summary table of decellularised tissue ECM-based biomaterials

Biomaterial Components	Form Factor	Decellularisation Method	In Vitro Characterisation	In Vivo Model	In Vivo Outcome	Reference
Human dermis	Scaffold	Peracetic acid solution	Endothelial cell and fibroblast attachment and infiltration ECM deposition Vascular network formation	N/A	N/A	[105]
Human amniotic membrane, reconstituted fibrin, HyA	Scaffold	Triton X-100 SDC Tris buffer Water	Fibroblast adhesion and proliferation Non-cytotoxic Increased non-fibrotic gene expression Increased ECM deposition	Full-thickness third-degree burn wounds in New Zealand white rabbits	Native tissue adhesion Complete epithelialisation Angiogenesis Comparable native skin architecture	[125]
Porcine small intestine submucosa	Scaffold	Mechanical separation from jejunum, tunica serosa and tunica muscularis externa	N/A	Full-thickness rat wound models	Wound integration and exudate absorbance Horny layer at day 28 Non-significant decrease in wound area Reduced inflammatory cell infiltration Collagen deposition	[127]
Human umbilical cord perivascular cell (HUCPVC)-seeded decellularised human dermis	Scaffold	Hypo- & hypertonic solution Triton X-100 Potassium chloride Tris buffer Serine protease inhibitor Peracetic acid solution DNase and RNAase	Enhanced proliferation and migration of HUVPVCs	Streptozotocin-induced diabetic, full-thickness rat wound model	Accelerated wound closure Collagen fibre deposition and granulation tissue formation after 7 days Complete and thicker re-epithelialisation after 14 days Enhanced vascularisation and VEGFR-2 expression	[106]
Amniotic membrane, chitosan	Scaffold	PBS Trypsin SDS Water	Enhanced cell viability	Streptozotocin-induced diabetic, full-thickness mouse wound model	Accelerated wound healing Thicker granulation tissue Increased early-stage inflammatory cell and fibroblast infiltration Increased neovascularisation Enhanced late-stage healing with hair follicles and sebaceous glands Enhanced collagen deposition and organisation	[124]
Porcine dermis or urinary bladder	Hydrogel	Mechanical delamination Trypsin Water Ethanol Triton X-100 in EDTA/Tris Peracetic acid	Fibroblast infiltration and proliferation on both hydrogels High myoblast viability and myotube formation on dermal hydrogel	Rat partial-thickness abdominal wall defects	CD68 + cell infiltration Defect remodelling and myogenesis at borders of defect Higher myogenesis in urinary bladder hydrogels	[108]
Human amniotic membrane	Scaffold	Ethanol Water Triton X-100 Lipase DNase	N/A	Full-thickness rat wound models	Reduced wound area Reduced white blood cell count Increased α-smooth muscle actin Reduced TGF-β1 expression	[118]

Decellularisation is typically achieved using chemical, physical and enzymatic protocols, usually involving a combination of multiple methods to ensure complete removal of the cellular components [102]. Chemical decellularisation includes a variety of treatments such as detergents which disrupt the cell membrane, for example sodium dodecyl sulphate (SDS), sodium deoxycholate (SDC), and Triton X-100. Enzymatic decellularisation is characterised by the cleavage of DNA, RNA and proteins, using nucleases, proteases, and trypsin [103]. Finally, mechanical decellularisation involves physically disrupting cell-ECM interactions through repetitive cycles of freeze-thawing, increasing hydrostatic pressure, cell scraping, and other disruptive treatments [100]. Although these methods remove much of the cellular components, they are typically insufficient on their own and require an additional decellularisation procedure for complete cell removal. Similarly, decellularised matrices should be evaluated post-treatment to ensure key structural integrity and mechanical properties have not been disrupted [103]. The decellularisation method used must be optimised depending on the tissue used, ensuring efficient retention of ECM components, given the potential for ECM disruption by stronger detergents [104].

Donor dermis sections are decellularised to form acellular dermal matrices (ADM), providing a template for cell infiltration and proliferation. For example, decellularised reticular dermal tissue has allowed for the infiltration of fibroblasts and endothelial cells, in addition to their deposition of key ECM components e.g., collagen IV [105]. Further, decellularised dermal tissue scaffolds have accelerated wound healing in a diabetic rat model, through increased re-epithelialisation and matrix deposition [106]. Upon delivery of human umbilical cord perivascular cells, re-epithelialisation and matrix deposition was further enhanced, in addition to the increased expression of vascular endothelial growth factor (VEGF) receptors and vascularisation [106]. ADMs may also be further processed and reconstituted into lyophilised sponges or injectable hydrogels. For example, pepsin digestion of the decellularised dermis, followed by induction of physiological pH and osmolarity stimulates hydrogel formation [107]. Porcine dermal hydrogels produced using this process have supported cell viability and infiltration, demonstrating their potential in DFU treatment [108]. Finally, cadaveric dermis-derived scaffolds have facilitated host cell invasion in a murine wound model, despite the fact that only 92.1% of DNA was removed during decellularisation [109]. Optimisation of the decellularisation protocol significantly improved DNA removal (99.8%) through the addition of a gamma irradiation step. Although gamma irradiation is widely reported to cause collagen crosslinking, basement membrane damage and radiation-induced fibronectin damage, there was no difference observed in mechanical properties, host cell infiltration, or collagen denaturation [110, 111].

ADMs have demonstrated clinical success in the treatment of diabetic wounds, with a number of studies reporting significant reductions in wound size, and healing times [112, 113]. Similarly, ADMs on the market including Alloderm™, DermACELL™, GraftJacket^®^, and AlloPatch^®^ offer a promising alternative to full-thickness skin grafts. Alloderm™, derived from cadaveric skin, has been used clinically for the treatment of full-thickness burns, showing decreased fibrosis and scar formation in comparison to other dermal substitutes [49, 114]. In a multicentre assessment of two human-derived ADMs, DermACELL™, and GraftJacket^®^, it was determined that DermACELL™ enhanced DFU healing in comparison to standard treatment, with 67.9% wound closure and 91.4% reduction in wound size [115]. Notably, this represented a significant improvement in DFU healing in comparison to previous studies [48, 114].

While the skin appears to be the most relevant tissue type from which to extract ECM and develop a biomaterial for wound repair, a number of other tissues have been assessed. This includes the amniotic membrane which has gained significant interest for tissue repair due to the abundance of HyA, collagens and laminins in its ECM [116]. Furthermore, the functions it serves *in utero*, (tissue development and growth, environmental protection, embryonic development, water retention, angiogenesis, and growth factor/nutrient transfer) closely align with the functions required of a biomaterial for wound repair [117]. The use of decellularised human amniotic membrane in the treatment of full-thickness rat wound models significantly reduced wound size. Similarly, VEGF and α-smooth muscle actin expression was enhanced, indicating the onset of the proliferative and remodelling phase respectively [118]. Biovance^®^ is a clinically approved, decellularised amniotic membrane-based wound dressing which is used for DFU treatment [119]. In the Letendre trial, Biovance^®^-treated DFU wounds decreased significantly in size over 12 weeks, with at least a 50% reduction in over 80% of participants, including complete closure in 55.5% of participants [120, 121]. Similarly, Omnigen, clinically approved for use in ocular wounds, has significantly reduced wound size when applied to patient DFUs, in comparison to standard care in DFU patients [122]. Epifix is a decellularised tissue matrix which is derived from sections of human chorion and amnion layers, which showed superior DFU healing in comparison to established ADMs [123]. Lastly, the combination of decellularised amnion and chitosan has facilitated proliferation of fibroblasts, in addition to the acceleration of diabetic wound healing, vascularisation, and ECM deposition in mouse models [124].

Despite promising results in decellularised amniotic membrane studies, there are drawbacks such as immunogenicity, difficult handling and manipulation, and the need for adhesives to secure the biomaterial in place. In order to partially overcome these challenges, Ramakrishnan and colleagues reinforced the mechanical properties of decellularised amniotic membrane through the addition of a fibrin-HyA scaffold layer. The scaffold which had increased thickness and tensile strength, was shown to facilitate fibroblast adhesion and proliferation, in addition to endothelial cell recruitment and reduced immunogenic effects [125].

Porcine SIS has also been extensively investigated for the treatment of DFU. Its ECM composition is similar to that of skin, with high collagen, elastin and glycosaminoglycan (GAG) content. Furthermore, it has been shown to facilitate cellular processes important for wound healing, including angiogenesis, cellular proliferation and differentiation [126]. Despite immediate inflammatory signs, the implantation of a SIS-derived sponge to a full-thickness wound in a rat model facilitated increased exudate absorption, re-epithelialisation and inflammatory resolution after 4 weeks, in comparison to established wound dressings [127]. The treatment of DFUs with Oasis^®^, a SIS-derived 3-layered decellularised matrix, reduced wound size, and aided complete wound healing in 54% of patients, compared to 32% of those treated with standard care [128]. Similarly, the treatment of pressure ulcers, another type of chronic wound, with Oasis^®^ significantly improved healing, with 55% reduction in wound size over 12 weeks. This surpassed the healing of standard care-treated wounds, which failed to exceed 40% incidence [129].

In summary, decellularised tissue-derived biomaterials have demonstrated significant promise in the treatment of DFUs and chronic wounds through the facilitation of increased wound healing, ECM deposition and re-epithelialisation. This is further emphasised by their approval and use in the clinic. However, important considerations to be made include optimisation of the decellularisation protocols to mitigate any potential immunogenic responses. In addition, while a wide range of tissue sources have demonstrated pro-regenerative effects, the optimal source has not been identified, and may differ depending on the type of wound and/or the precise anatomical location.

### In vitro-generated ECM biomaterials

Biomaterials generated from in vitro-deposited ECM constitute an innovative approach to wound healing. Two main approaches have been explored, involving the decellularisation and collection of ECM deposited by cells in 2D culture and its incorporation within a biomaterial, or direct deposition of ECM by cells seeded within a pre-existing biomaterial structure, followed by cell removal [16]. There are many benefits to these approaches, particularly their tunability and specificity. For example, the selection of relevant cell types, control over the microenvironment, and stimuli which cells are exposed to, allows for optimisation of the ECM deposition. This level of control is not possible in other methods including tissue decellularisation, as previously outlined [130].

For the production of in vitro-generated ECM biomaterials, cell monolayers are decellularised for the preservation of the deposited ECM, followed by ECM collection and incorporation within a biomaterial [131]. For example, ECM deposited by human induced pluripotent stem cell (hiPSC)-derived fibroblasts was decellularised and subsequently incorporated into a collagen-GAG scaffold by lyophilisation. Higher anti-inflammatory M2 macrophages and VEGF expression was observed in ECM-functionalised scaffolds, indicating their promise to direct the resolution of inflammation and enhance vascularisation [132]. It is also interesting to note the greater capacity of ECM deposited from hiPSC-derived fibroblasts to drive pro-reparative processes in comparison with ECM derived from adult fibroblasts [132]. These effects may be related to the apparent ‘rejuvenation’ of cells, closely resembling their foetal counterparts, thus promoting scarless healing [93]. In another pre-clinical study, scaffolds were produced combining decellularised lung fibroblast-derived ECM and collagen type I. The application of this scaffold in diabetic wound models showed great potential for DFU healing, with higher rates of wound closure, improved vascularisation, and increased collagen deposition in vivo [133]. While these studies emphasised the potential of the technique in DFU therapeutics, they also indicate areas in which the technique can be further enhanced. For example, optimisation of the cellular niche through manipulation of the cellular microenvironment may induce more potent effects on processes related to wound healing such as angiogenesis. For example, hypoxic conditions in human dermal fibroblast cultures significantly increased ECM-deposition in vitro, in addition to increased endothelialisation of cells on a polycaprolactone (PCL) graft, VEGF, and angiotensin-1 expression [134].

Another approach involves the direct seeding of cells on or in an existing biomaterial, in which they deposit ECM over time. Subsequently, decellularisation produces an acellular construct which is laden with cell-deposited ECM. In a comparative study, patient-derived iPSC fibroblasts induced similar ECM deposition to 2D seeding, with enhanced GAG deposition and vascularisation when seeded to a scaffold [135]. Similarly, mesenchymal stromal cells seeded on silk fibroin scaffolds and decellularised, enhanced wound healing in diabetic mouse models. This was demonstrated by reduced wound size over 10 days, increased migration of human umbilical vein endothelial cells (HUVECs) and related expression of angiogenic growth factors, in comparison to silk fibroin controls [136]. However, the decellularisation of pre-optimised scaffolds raises a concern of compromised mechanical properties and structural properties following decellularisation. Thus, care must be taken to optimise the decellularisation process to avoid damaging the structural integrity of the scaffold. To overcome this, ECM-derived scaffolds have been produced using a poly(lactic-co-glycolic) acid template, which was removed after the decellularisation process. This allowed for the formation of pure ECM scaffolds which facilitated high fibroblast viability, attachment, and proliferation indicating their potential in skin regeneration [137].

Although the number of skin-related studies is limited, the success of in vitro-generated ECM biomaterials in the regeneration of other tissues can be extrapolated and applied to the development of DFU therapeutics in the future. For example, Carvahlo et al. seeded co-cultures of MSCs and HUVECs on ECM/PCL electrospun scaffolds for bone tissue engineering. The combination of lyophilised MSC- and HUVEC-ECM with polycaprolactone (PCL) facilitated enhanced osteogenesis and angiogenesis simultaneously [131]. This indicates the potential of this approach, which may be adapted and harnessed for the treatment of DFUs.

Finally, a major limitation of cell-derived ECM is the duration of culture required to produce a sufficient yield. As such, a range of approaches are being explored to enhance and accelerate ECM deposition. For example, macromolecular crowding is a particularly promising technique based on the excluded-volume effect, such that the addition of an inert macromolecule enhances ECM deposition [138]. Ficoll and carrageenan have been identified as promising macromolecules to enhance ECM deposition of iPSC-derived fibroblasts and human adipose-derived stem cells in vitro, and have subsequently been used to produce ECM-derived biomaterials that support healing in murine wound models [139, 140]. Similarly, addition of ascorbic acid to culture media facilitates ECM deposition through the upregulation of collagen gene transcription and synthesis, and inhibition of their degradation [141]. A summary of in vitro generated ECM-derived biomaterials is depicted in Table 3.

**Table 3:** Summary table of in vitro generated ECM-derived biomaterials

Biomaterial Components	Form Factor	Decellularisation Method	In Vitro Characterisation	In Vivo Model	In Vivo Outcome	Reference
Fibroblast-deposited ECM, collagen	Scaffold	0.25% Triton X-100 Ammonium hydroxide DNase & RNase	Increased fibroblast migration α-SMA negative quiescent fibroblasts ECM deposition Upregulation of tissue remodelling markers	Full-thickness wounds, leptin receptor *db/db* mice	Increased wound healing, blood vessel area, remodelling and hair follicles Increased wound closure High mature blood vessel area Increased collagen deposition Reduced dermal/epidermal thickness	[133]
Fibroblast-deposited ECM, collagen, chondroitin-6-sulphate	Scaffold	Water Snap-freeze Lyophilisation	Extended healthy and DFU fibroblast proliferation and GAG deposition Increased ECM deposition and component variety of all ECM-derived scaffolds Enhanced inflammatory response	N/A	N/A	[132]
Patient-derived fibroblast- deposited ECM, collagen, chondroitin-6-sulphate	Scaffold	Water Freeze-thaw DNase	Enhanced healthy and patient-matched DFU fibroblast proliferation Increased vessel area and GAG deposition in decellularised scaffold	N/A	N/A	[135]
Mesenchymal stromal cell-deposited ECM, silk fibroin	Scaffold	Water	Enhanced HUVEC migration and angiogenic factor secretion	Punch biopsy wounds on leptin receptor *db/db* mice	Improved wound healing of 49 ± 0.8% compared to 50 ± 2.4% in not decellularised control in 3 days Increased expression of angiogenic and remodelling genes	[136]
Fibroblast-deposited ECM within Poly (lactic-co-glycolic acid) scaffold	Scaffold	Freeze-thaw Ammonium hydroxide	High fibroblast viability Even fibroblast distribution Dermal regeneration	N/A	N/A	[137]

## Mechanisms of action

**Fig. 3:**
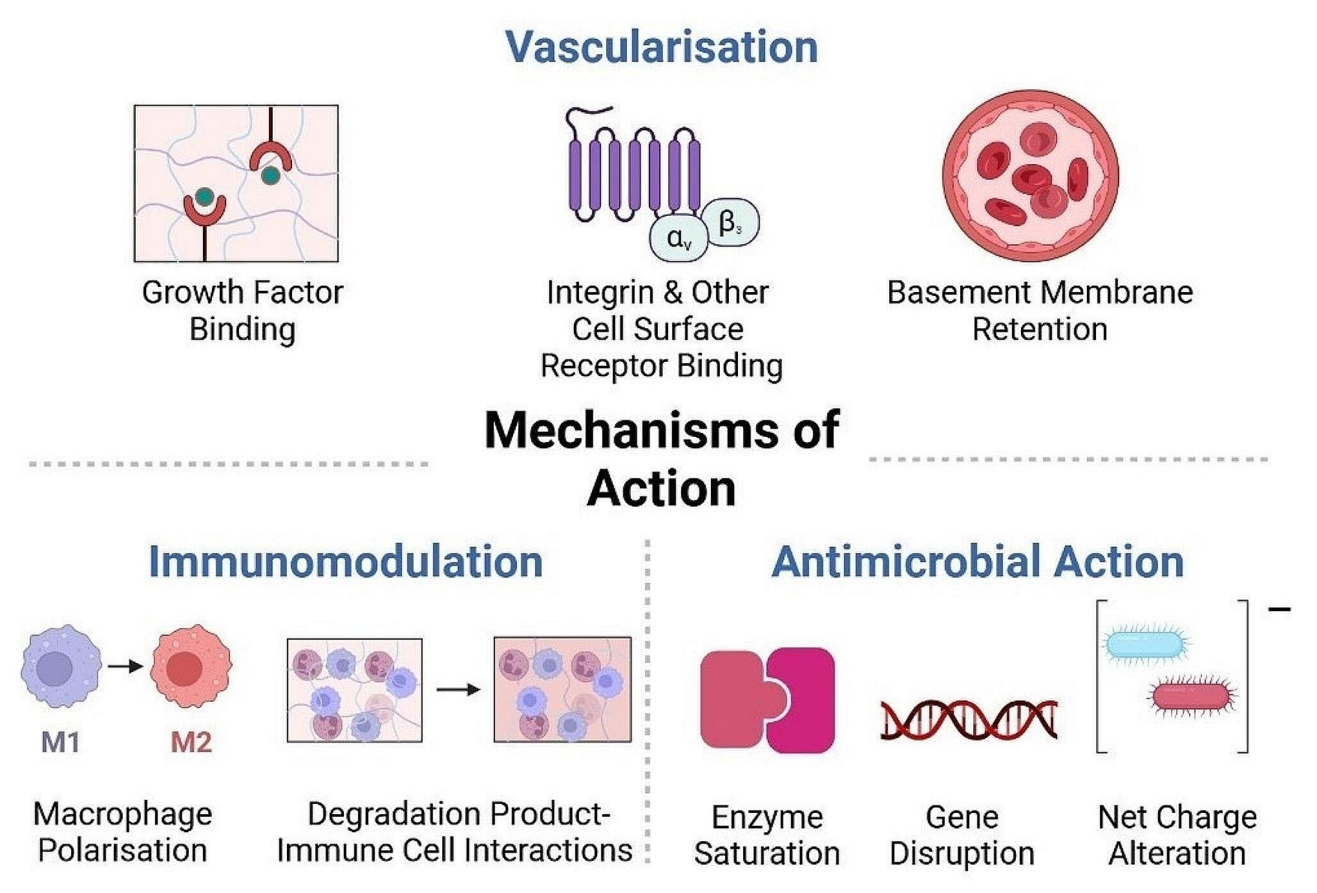
The mechanisms of action through which biomaterials stimulate wound healing consist of angiogenesis, immunomodulation, and antimicrobial action. Through growth factor binding, integrin and other cell-surface receptor binding, and the retention of basement membrane proteins and architecture, biomaterials stimulate angiogenesis. Through macrophage polarisation, and cellular interactions with biomaterial degradation products, biomaterials elicit immunomodulatory effects. Finally, through the inhibition of hyaluronidase, gene disruption and alteration of the bacterial cell wall net charge, biomaterials can induce wound healing through antimicrobial action. Figure was created in Biorender

ECM-derived biomaterials have demonstrated great promise for tissue repair in a wide range of applications including the treatment of chronic wounds. A summary of the clinical studies associated with many of these ECM-derived biomaterials can be seen in Table 4. In order to fully harness the promise of ECM-derived biomaterials, it is essential that the mechanisms of action of these materials are fully understood. Generally, biomaterials aim to support tissue repair by providing a structure or template that supports host cell infiltration and proliferation. In addition to this, ECM-derived biomaterials support tissue repair through a number of mechanisms associated with their inherent bioactivity, including immunomodulation, promotion of vascularisation and antimicrobial activity. Each of these mechanisms, and how they relate to wound repair, are discussed below, with a visual summary in Fig. 3.

**Table 4:** Summary table of ECM-derived biomaterials clinical studies

Biomaterial Components	Form Factor	Patient Cohort	Clinical Outcome	Reference
**Purified ECM Component Biomaterials**				
Collagen, Chondroitin-6-Sulphate, Silicone	Scaffold	DM patients with ≥ 1 DFU with grade 1/2 Wagner classification	Accelerated wound closure	[48]
Collagen, Chondroitin-6-Sulphate, Silicone	Scaffold	Full-thickness traumatic wounds	78% wound closure in treatment group	[91]
Collagen, Chondroitin-6-Sulphate, Silicone	Scaffold	Patients with surgically excised burn wounds	Comparable vascularisation and scaffold anchorage to wound for epidermal growth	[92]
Collagen, Chondroitin-6-Sulphate, Silicone	Scaffold	Acute and chronic burn patients	88% full restoration of articular motion and flexible skin coverage	[93]
Collagen, Elastin	Scaffold	Type-2 diabetic patients with chronic DFUs	Shorter hospitalisation period and accelerated healing	[94]
Collagen, Elastin	Scaffold	Traumatic skin loss recovery in a diabetic, hypertensive, and ischemic heart disease patient	Reduced loss of substance, complete closure, decreased limb oedema, functional rehabilitation	[96]
Collagen, Oxidised Regenerated Cellulose	Scaffold	DM patients with DFU for ≥ 6 weeks	Reduced level of MMP-2, gelatinase, elastase in wound exudate and reduced wound size	[60]
Collagen Type I	Particles	Patients with non-healing ulcers without bone exposure	37.29% wound reduction compared to 14.29% in the control group	[61]
Platelet Rich Fibrin	Combined platelet-derived scaffold and injectable PRP gel	Diabetic and non-diabetic advanced wound patients	Diabetic patient wound size reduced to less than 25% after 4 weeks Enhanced wound healing in both diabetic and non-diabetic patients	[82]
Platelet Rich Fibrin	Patch (Autologous fibrin)	DM patients with ≥ 1 DFU and a baseline HbA1c of ≤ 108 mmol/mol	Accelerated and enhanced wound healing	[83]
Platelet Rich Fibrin, HyA	Hydrogel	DM patients with 3-month duration DFU with grade 2 Wagner classification	Increased VEGF and decreased IL-6 after 7 days	[98]
Collagen, Chondroitin-6-Sulphate	Hydrogel (Granular collagen mixed with saline)	Diabetic patients with DFUs with grade 3 Wagner classification	Accelerated and 86.95% complete healing in treatment group	[90]
Collagen, Chondroitin-6-Sulphate	Hydrogel (Granular collagen mixed with saline)	Diabetic patients with DFUs with grade 3 Wagner classification	Decreased infection, accelerated healing compared to IDRT, reduced expression of inflammatory biomarkers, increased expression of ECM remodelling and angiogenesis	[89]
HyA	Scaffold	Diabetic patients with DFUs with grade 1/2 Wagner classification	65.3% complete healing compared to 49.6% in control group	[64]
HyA	Scaffold	Diabetic patients with DFUs for ≥ 1 month	Accelerated wound closure in treatment group	[75]
**Decellularised Tissue Biomaterials**				
Decellularised human dermis	Scaffold (Chemical and enzymatic decellularisation, and gamma irradiation)	Volunteer full-thickness punch biopsy wounds	Increased dermal thickness, reduced dermal fibrosis	[114]
Decellularised human dermis	Dressing (Chemical decellularisation and lyophilisation)	Diabetic patients with a 1 full-thickness DFU with Wagner classification of 1 or 2	Higher complete healing rate than control, reduced wound area compared to all groups	[115]
Decellularised human dermis	Hydrogel (Dermis in aqueous gelatin solution)	Patients with chronic full-thickness wounds which failed to heal over 3 weeks	76.3% complete healing in 12 weeks, accelerated granulation and re-epithelialisation	[112]
Decellularised human dermis	Hydrogel (Dermis in aqueous gelatin solution)	Diabetic patients with DFUs with Wagner classification grade 2 or 3 for at least 4 weeks	56.52% complete healing over 60 days compared to 23.08% in control group, accelerated wound healing compared to control	[113]
Decellularised human amniotic membrane	Scaffold (Ocular ulcer treatment) (Chemical decellularisation)	Diabetic patients with ≥ 1 DFU	27% healing compared to 6.3% in control group, higher wound area reduction	[122]
Porcine small intestine submucosa	3-Layered Scaffold (Chemical decellularisation)	Diabetic patients with ≥ 1 DFU for > 6 weeks to 1year	54% healing compared to 32% in control group, higher wound area reduction at all visits	[128]
Decellularised human dermis	Scaffold (Chemical decellularisation)	Full-thickness burn patient	Keratinocyte and fibroblast infiltration, neovascularisation, wound re-epithelialisation, increased dermal elasticity	[49]
Decellularised human amniotic membrane	Scaffold (Chemical decellularisation)	DM patients with chronic DFUs with Wagner classification of 1 or 2	55% re-epithelialisation, 50% reduction in wound size in 33.3% of patients	[121]

### Immunomodulation

Inflammation and its timely resolution are crucial to DFU healing, and tissue repair in general. As an initial response to injury, inflammation plays a key role in initiating the repair response, ensuring the removal of necrotic debris and invading microbes [142]. However, in compromised wound healing and chronic wounds such as DFUs, the inflammatory response fails to resolve in a timely manner, manifested by the persistence of M1-type macrophages in the wound. In a healing wound, alteration of the macrophage phenotype from M1 to M2 shifts the balance of the wound microenvironment from pro-inflammatory to anti-inflammatory, supporting inflammatory resolution and wound healing [25]. ECM-derived biomaterials have been shown to play a key role in this process, displaying an innate capacity to drive macrophage polarisation towards a pro-healing phenotype [18]. In the context of ECM-derived biomaterials, an inflammatory cascade is initiated after scaffold implantation to a wound site. This includes infiltration of a variety of immune cells including macrophages and T-cells, which stimulate paracrine signalling and macrophage polarisation [143]. However, there are many contributary factors which may affect the immune response to ECM biomaterials. This can include the degradation products of the material, its tissue origin, and the efficiency of decellularisation.

Degradation products play a key role in ECM biomaterial-driven immunomodulation as the interactions between degradation products and immune cells (including T-regulatory, T-helper cells, and macrophages) drives the downstream immune reaction [144]. Studies have investigated the potential of ECM-derived biomaterials and their degradation products to promote skeletal muscle regeneration and induce macrophage polarisation. After treatment with SIS-ECM degradation products, a high frequency of Fizz1, a marker for M2 activation, was detected in bone marrow derived macrophages, indicating the anti-inflammatory immunomodulatory capacity of SIS-ECM [145]. Similarly, the ECM tissue-of-origin contributes to macrophage polarisation, as seen by the differential capacity of decellularised tissues to induce M2 polarisation in vitro, with the M2 phenotype potently driven by SIS, bladder, and colonic tissue ECM-derived scaffolds. Interestingly, a pro-inflammatory effect was seen after treatment with dermal-ECM, represented by iNOS+, Fizz1-, and cluster determinant molecule (CD)-206 expression [146].

Furthermore, the efficacy of the decellularisation protocol is a key determinant of the immunomodulatory capacity of a decellularised tissue-derived biomaterial. Remnants of foreign body antigens can stimulate the persistence of a pro-inflammatory, non-healing, M1 phenotype [147]. In contrast, more intensive or thorough methods of decellularisation, elicit a predominant M2 phenotype in decellularised SIS-derived biomaterials [148]. Moreover, it has been recently determined that the implantation of decellularised tissue ECM-derived biomaterials can drive the foreign body response towards a modulatory phenotype. Decellularised cardiac muscle and bone ECM-based biomaterials stimulated T-helper cell and IL-4 driven macrophage M2 transition, initiating paracrine signalling, remodelling and healing in a volumetric muscle loss model [149]. Similarly, a porcine SIS-derived material induced a substantial reduction of classical Inflammatory Bowel Disease markers in a murine model. This occurred by modulation of the M1 macrophage polarisation and reduction in TNF-α expression [150].

Specific purified ECM components including HyA have also been shown to induce M2 macrophage polarisation [18]. High molecular weight HyA in particular, has been shown to drive M2 macrophage polarisation, which is associated with the expression of anti-inflammatory cytokines such as IL-10 [151]. When high molecular weight HyA binds to CD44, phagocytosis is initiated, promoting the timely resolution of inflammation, and healing [152]. This effect can be further enhanced through functionalisation of HyA through the addition of sulphate groups. This was seen through the regulation of oxidative stress and upregulation of associated antioxidants such as superoxide dismutase (SOD)-2 and − 3 [153]. The anti-inflammatory effects of HyA were further demonstrated by a sulphated, high molecular weight HyA/Collagen hydrogel. In a diabetic mouse model, it was observed that wounds which were treated with the sulphated HyA/Collagen hydrogel had lower expression of pro-inflammatory genes including IL-1β and NLR family pyrin domain containing 3 (NLRP3), higher expression of IL-10, and higher rates of wound healing, in comparison to non-sulphated controls [154].

The unique capability of ECM-derived biomaterials to tune the immune response during wound healing is key factor which makes them particularly useful in DFU therapeutics. The interactions which occur between immune cells, biomaterials and their degradation products, can drive immunomodulation through mitigation of the foreign body response and macrophage polarisation.

### Vascularisation

Vascularisation of the wound bed, and the restoration of oxygen/nutrient delivery, is critical to wound healing [155]. In general, the ECM contains an abundance of pro-angiogenic components, growth factors, and signalling molecules which support and/or promote vascularisation through integrin-mediated endothelial cell-matrix interactions [156]. In particular, α_v_β_3_ and α_v_β_5_ integrins expressed by endothelial cells, play key roles in vascularisation through the promotion of endothelial cell proliferation [157]. Similarly, ECM components play a key role in growth factor retention and signalling to promote vascularisation. Fibroblast growth factor (FGF) for example, is a known pro-angiogenic growth factor that binds to heparan sulphate present in the ECM and propagates downstream signalling required for endothelial cell proliferation, migration and blood vessel formation [158]. For example, perlecan, a heparan sulphate proteoglycan typically present in basement membranes, facilitates angiogenesis through FGF and VEGF binding [159]. Conjugation of heparan sulphate to collagen-based biomaterials enhances growth factor retention within the hydrogel, supporting blood vessel formation as observed in the chorioallantoic membrane assay [160].

ECM-derived biomaterials in general, have shown significant angiogenic potential, for example, purified ECM component biomaterials containing fibrin, HyA, collagens, and gelatin. The presence of α_v_β_3_ and heparin binding domains on fibrin allows for cell attachment, biomaterial infiltration, and subsequent angiogenesis [161]. Similarly, in fibrin-based biomaterials, plasmin-mediated fibrinolysis and the breakdown of the fibrin complex forms multiple fragments, with fragment E playing a pro-angiogenic role in wound healing [162]. A number of in vivo studies have demonstrated the ability of fibrin and collagen hydrogels to facilitate angiogenesis in ischemic tissue, for example in a rat model of myocardial infarction [163].

Further, low molecular weight HyA is known to initiate neo-vascularisation through cell surface receptor binding, initiation of the inflammatory cascade, and eventual endothelial cell proliferation [164]. Cell surface receptors such as CD44 and receptor for HA-mediated motility (RHAMM) bind to HA oligomers, thus inducing mitogenesis and endothelial cell proliferation through mitogen-activated protein (MAP) kinase signal transduction [165]. The modification of HyA-based biomaterials is a common approach to the stimulation of angiogenesis/vascularisation. For example, the addition of RGD binding sites to HyA has facilitated increased vessel lumen formation, neovascularisation, and vascular patterning in vitro [166]. Similarly, the delivery of pro-angiogenic growth factors including VEGF and PDGF (platelet-derived growth factor) has been possible through their adhesion to binding sites on laminin (multiple isoforms)-fibrin based biomaterials. The delivery of these growth factors through heparin binding domains on α-chain laminin-type G and their inclusion in fibrin matrices, successfully promoted wound healing in DFU mouse models [167].

Decellularised tissue matrices similarly provide a platform to promote angiogenesis. Through the retention of ECM components and growth factors, decellularised tissues can stimulate pro-angiogenic processes [168]. Interestingly, the decellularisation protocol used can also have a potent effect on the formation of new vessels. This includes maintaining the architectural integrity of the ECM, allowing for effective recellularisation and vascularisation [169]. Similarly, retention of the vascular basement membrane proteins can be a critical predictor of the angiogenic capacity of decellularised tissue-derived ECM biomaterials, due to their abundance of collagens, fibronectin, proteoglycans etc. [170]. This was further seen in vitro, with immobilised and aligned ECM components mimicking the basement membrane. The aligned ECM protein components facilitated the formation of new blood vessels through HUVEC adhesion and proliferation [171]. Finally, the incorporation of cell-derived ECM has displayed pro-angiogenic potential when incorporated in collagen scaffolds. HUVEC seeding on iPSC-derived fibroblast ECM-collagen-GAG scaffolds resulted in increased tube formation and vascularisation, indicating their pro-angiogenic potential in wound healing [135].

### Antimicrobial action

The non-healing nature of DFUs is exacerbated by the presence of bacterial or microbial colonies which elevate and extend the inflammatory response [120]. Although limited, there is some evidence supporting the antimicrobial action of ECM components, most notably HyA [172]. In a dose- and molecular weight-dependant manner, reduced growth of *S. aureus* and various other bacterial pathogens, has been demonstrated on HyA-based biomaterials, in comparison to biomaterials which are commonly used in orthopaedic implants [173]. Although yet to be fully elucidated, one proposed antimicrobial mechanism of HyA is related to bacterial synthesis of hyaluronidase. Normally, bacterial synthesis of hyaluronidase facilitates local degradation of HyA, allowing bacterial colonisation and infection within a wound. However, it has been speculated that the presence of a higher concentration of HyA saturates the hyaluronidase enzyme, thus reducing bacterial hyaluronidase activity, bacterial proliferation, and preventing attachment within the wound site [174]. Similarly, the amide and carboxyl groups present on the HyA macromer elicit a combined negative charge that repels the negatively charged bacterial cell wall, and prevents the attachment of microbes [175]. Liu et al., fabricated a HyA-based hydrogel which was enzymatically crosslinked with Ɛ-polylysine. This combination of HyA and Ɛ-polylysine effectively reduced the amount of *E. coli* and *S. aureus* in infected rat wounds in this instance [176].

Similarly, other ECM-components including laminin and fibronectin have also been reported to elicit antimicrobial activity through bacterial DNA disruption. Through heparin-binding, several α4 and α5 laminin peptide chains initiate membrane lysis and DNA destruction of *E. coli* and *S. aureus* [177]. Finally, the collagen IV α_3_ subunit causes intracellular damage to bacteria including *S. aureus* by inducing damage to the extracellular membrane and releasing the cellular contents [178]. The innate antimicrobial capacity of biomaterials can be further enhanced through several methods including surface modifications, pH manipulation, and the addition of specific antimicrobial agents. For example, the addition on chitosan, a natural polysaccharide derived from crustaceans with known antimicrobial activity, or conjugation of antimicrobial peptides (AMPs) [179–181].

## Discussion

Although the current standard of care for diabetic wounds contains rigorous protocols for wound dressing, antibiotic treatment, and pressure off-loading, these approaches are insufficient to resolve the chronic wounds of diabetic patients. This is evident from the frequency of limb amputations worldwide as a direct result of DFUs. In recent years, the growing potential of biomaterials for the treatment of DFU has come to the fore, with a particular focus on the development of ECM-derived biomaterials [13]. The ECM-derived biomaterials discussed within this review include biomaterials composed of isolated ECM components, and the combination of multiple components. Similarly, the decellularisation of tissues, and the incorporation of in vitro-deposited ECM in biomaterials, allows for the inclusion of a full biological repertoire of ECM components within a biomaterial, similar to that of the native tissue. These ECM-derived biomaterials support wound healing through vascularisation, immunomodulation, and protection against microbes, in comparison to the current standard of care.

While this review discusses a number of highly promising studies, further optimisation of decellularisation techniques is necessary to fully understand and harness the potential of ECM-derived biomaterials in wound healing. For example, in vitro cell-deposited ECM is a relatively recent and novel technique and is less studied as a result. Similarly, future studies should focus on the functionalisation of ECM-derived biomaterials to further support their therapeutic effect, including the addition of therapeutic agents to further their inherent capacity to drive immunomodulation, angiogenesis, and antimicrobial action [155, 182, 183]. Recent examples of this include the loading of decellularised ovine pericardial-derived scaffolds with resveratrol, which enhanced their antibacterial, swelling, and water retention capacities [184]. Similarly, the addition of zinc oxide and vitamin c to decellularised caprine small intestine submucosa hydrogels enhanced burn wound healing through increased wound contraction, reduced inflammatory cell infiltration, and increased collagen deposition in rabbit models [185].

Other potential avenues for the optimisation and understanding of ECM-derived biomaterials for wound healing include their assessment in microphysiological systems, or so-called organs-on-a-chip [186]. The development of microfluidic systems to interrogate the bioactivity and regenerative capacity of ECM-derived biomaterials using human cells would allow for more high throughput analysis of ECM-derived biomaterials in more physiologically relevant systems with human cells and incorporation of biophysical stimuli such as fluid flow [187]. Similarly, it would facilitate more accurate assessment of pro-angiogenic biomaterials and the vasculature in vitro, through the induction of biophysical signals such as shear stress, fluid flow, offering efficient, reproducible, and complementary systems to preclinical animal testing [188, 189]. For example, the pro-angiogenic capacity of human fibroblast-deposited ECM/ collagen type I composite hydrogels has been assessed in microfluidic devices that incorporate fluid flow [190]. This type of high throughput system can act as a bridge to refine and optimise ECM-derived biomaterials prior to preclinical studies, reducing the use of animal models while also providing a 3D test-bed that recapitulates critical aspects of human physiology.

Moreover, through analysis of reported healing rates, it has become evident that DFU severity is heterogenous due to a variety of contributing factors including patient age, overall health, and lifestyle, thus preventing a ‘one size fits all’ solution [6]. Incorporating organ-on-a-chip and advanced stem cell technologies in DFU research has the potential to facilitate the development of disease or even patient-specific or personalised medicine approaches, and the stratification of patients by wound severity using ‘on-chip’ physiology. The incorporation of patient-specific cells would facilitate a deeper understanding of the cellular processes within the DFU of a specific patient, and how ECM components and complementary therapeutics could contribute to its healing through inflammatory resolution, angiogenesis, and antimicrobial action.

## Concluding remarks

ECM-derived biomaterials show great promise to improve on the current standard of care for the treatment of DFU. However, a number of hurdles must be overcome before this becomes a reality. This includes the optimisation of tissue sources, and approaches to their subsequent processing and biomaterial development, which are yet to be defined. The biomaterials discussed within this review incorporate the ECM in a variety of ways, ultimately aiming to develop regenerative biomaterials to support healing. In this way, ECM-derived biomaterials stimulate processes such as immunomodulation, angiogenesis, and antimicrobial action to drive wound healing. The translation of these biomaterials to pre-clinical and clinical DFU studies will reduce the burden of DFUs, ultimately restoring some quality of life to DM patients.

## Data Availability

No datasets were generated or analysed during the current study.
